# The Antiedematous Effect of Exogenous Lactate Therapy in Traumatic Brain Injury: A Physiological and Mechanistic Approach

**DOI:** 10.1007/s12028-021-01219-y

**Published:** 2021-04-20

**Authors:** David Emmanuel Duhaut, Catherine Heurteaux, Carine Gandin, Carole Ichai, Hervé Quintard

**Affiliations:** 1 Intensive Care Unit, Hospital Pasteur 2, Le Centre Hospitalier Universitaire de Nice, Nice, France; 2 UMR7275, Institut de Pharmacologie moléculaire et cellulaire, Valbonne, France; 3 Intensive Care Unit, Hôpitaux Universitaires de Genève, Geneva, Switzerland

**Keywords:** Lactate sodium, Brain edema, Aquaporin, K–Cl transporter

## Abstract

**Background:**

Sodium lactate (SL) has been described as an efficient therapy in treating raised intracranial pressure (ICP). However, the precise mechanism by which SL reduces intracranial hypertension is not well defined. An antiedematous effect has been proposed but never demonstrated. In this context, the involvement of chloride channels, aquaporins, or K–Cl cotransporters has also been suggested, but these mechanisms have never been assessed when using SL.

**Methods:**

In a rat model of traumatic brain injury (TBI), we compared the effect of SL versus mannitol 20% on ICP, cerebral tissue oxygen pressure, and brain water content. We attempted to clarify the involvement of chloride channels in the antiedematous effects associated with lactate therapy in TBI.

**Results:**

An equimolar single bolus of SL and mannitol significantly reduced brain water content and ICP and improved cerebral tissue oxygen pressure 4 h after severe TBI. The effect of SL on brain water content was much longer than that of mannitol and persisted at 24 h post TBI. Western blot and immunofluorescence staining analyses performed 24 h after TBI revealed that SL infusion is associated with an upregulation of aquaporin 4 and K–Cl cotransporter 2.

**Conclusions:**

SL is an effective therapy for treating brain edema after TBI. This study suggests, for the first time, the potential role of chloride channels in the antiedematous effect induced by exogenous SL.

## Introduction

Brain edema is a major complication of traumatic brain injury (TBI). It increases the risk of intracranial hypertension (ICH) and brain hypoxia, leading to an increase in mortality and poor neurologic outcome. Increased water content in the injured brain can be related to a vasogenic or cellular pathway. Osmotherapy, by using mannitol or hypertonic saline (HS), is recommended and currently administered for the treatment of ICH in this setting [1]. Beside these two usual treatments, sodium lactate (SL), a metabolic and neuroprotective solution [2–5], has recently been described as having similar effects on lowering intracranial pressure (ICP) [6, 7]. In a previous study, conducted in patients with severe TBI, Ichai et al. [6] reported that a bolus of half-molar SL was more effective than equimolar doses of mannitol to reduce elevated ICP (less refractory ICH and higher and longer reduction of ICP). In another clinical trial, a continuous infusion of SL over the first 48 h was shown to prevent by 50% the number of raised ICP episodes after severe TBI [7]. Although mannitol was responsible for plasma hyperosmolarity, no significant increase in plasma osmolarity was observed following SL perfusion. Moreover, in these studies, chloride balance in the SL-treated group was equivalent to that in the sodium chloride-treated group, suggesting that chloride excretion is possibly improved with SL infusion. Consequently, other mechanisms than those associated with a purely osmotic effect might be present.

Chloride channels are involved in a wide range of biological functions, including cell volume regulation and neuroexcitation. Mammalian chloride channels broadly fall into five classes on the basis of their regulation [8]. Of these, cation-chloride cotransporters (CCCs)—including the Na–K–2Cl cotransporter NKCC1, which mediates intracellular chloride influx, and the K–Cl cotransporters KCC2 and KCC3, which extrude chloride—play a major role in cell volume regulation. CCCs are expressed in mature neurons, glia, and the spinal cord [9]. The importance of an equilibrium in CCCs is evidenced by the functional alteration in intracellular chloride concentration seen in both NKCC1 and KCC2 knockout animals [10–12]. In addition, NKCC1 has been shown to play a crucial role in the mediation of astrocyte swelling and in brain edema. Oxidative stress after TBI or ischemia is responsible for NKCC1 phosphorylation (activation) and membrane translocation, resulting in increased Na^+^, K^+^, Cl^−^ intracellular influx, along with water intake and cell swelling [13]. In vitro experiments showed that NKCC1 expression significantly increased after fluid percussion on cultured astrocytes, whereas trauma-induced astrocyte swelling was significantly reduced by the administration of bumetanide, an inhibitor of NKCC1 [14]. Concurrently, cell swelling is countered by regulatory volume decrease, which involves the cellular loss of chloride and potassium via the activation of KCC [9]. Aquaporin (AQP) is also a major group of water regulation proteins. More than ten subtypes of AQP have been identified. Among them, aquaporin 4 (AQP4) has dual roles during the cerebral edema time course [15]. AQP4 inhibition during the early stages of TBI is protective against edema formation. However, AQP4 also plays a key role in water clearance from the brain to blood vessels during the later phase of trauma [16].

With a better understanding of the mechanisms involved in controlling brain edema, we could potentially expect to identify new treatments for ICH. On the basis of these observations, we designed a rat model of TBI to assess the effect of exogenous SL on reducing brain edema and also to explore the potential mechanisms that an SL infusion exerted on chloride channels.

## Methods

### Experimental Procedures

All experiments were performed on male Sprague Dawley rats (300–330 g) 7 weeks old and obtained from Janvier Laboratories (Saint-Quentin-Fallavier, France). Experiments were conducted according to the directives on the care and use of laboratory animals published by the European Communities Council Directive (2010/63/EU). The French ministry of higher education and scientific research approved the experiments (protocol number APAFIS#3550-201610618323927v3), and all efforts were made to minimize animal suffering and reduce the number of animals used.

### Lateral Fluid Injury Model

As previously described [17], we used a lateral fluid percussion injury (LFPI) model to create a TBI of high severity [18]. Briefly, rats were maintained under anesthesia by inhalation of 2% isoflurane and placed on a stereotaxic frame. Normothermia was maintain with a heating pad. Respiration rate and the depth of anesthesia were monitored continuously. We performed a 3-mm craniotomy on the right parietal cortex, 3.6-mm posteriorly and 3-mm laterally to the bregma, taking care to leave the dura mater intact. A plastic female luer lock disc was fixed to the craniotomy site by using cyanoacrylate adhesive and dental cement. After filling the luer lock disc with saline serum to control the sealing, anesthesia was stopped, and the animal was connected to the trauma device. LFPI was induced by using a device (Fluid Percussion Injury Device Model 01-B; Custom Design & Fabrication, Sandston, VA) consisting of a Plexiglas, cylindrical, saline-filled reservoir closed at one end by a Plexiglas plunger mounted on O-rings. The opposite end of the cylinder was capped with a male luer stub, which was connected to the rat via the female luer lock fitting. A pendulum was allowed to drop, striking the plunger and producing a calibrated outflow pressure pulse of 2.5 atm and of 20 ms in duration via the rapid injection of saline into the closed cranial cavity. The applied cortical pressure was measured extracranially by a pressure transducer connected to an oscilloscope. The luer lock fitting was then removed, and the scalp was sutured under anesthesia. Injection treatment was commenced 1 h after trauma according to previous data (results not shown). On the first hour after trauma, the rats received an intravenous bolus of either 1.5 ml/kg body weight of isotonic saline solution (vehicle group), 1.5 ml/kg body weight of 20% mannitol (mannitol group), or 1.5 ml/kg body weight of half-molar SL (SL group). Half-molar SL was designed to have a similar osmolarity to 20% mannitol and contained 504 mM Na, 4 mM K, 1.36 mM Ca, 6.74 mM Cl, and 504.1 mM lactate [6]. Moreover, in a preliminary experimental study assessing SL dose response, we did not find any therapeutic benefits to increasing SL concentration (results not shown). The control group consisted of Sham-operated rats that underwent the same surgery but without percussion. At the end of the experiment, animals were transferred to their home cages with free access to water and food.

### Physiological Measurements (Mean Arterial Pressure, ICP, and Cerebral Tissue Oxygen Pressure)

Prior to the surgical procedure previously described, a 25-gauge catheter was placed in the left femoral artery of the rat under inhaled anesthesia (*n* = 5 rats per group) for continuous mean arterial pressure monitoring. After trauma, the animal was transferred to a stereotaxic frame and maintained under inhaled anesthesia. An ICP probe (Transonic Science PV Catheter; EMKA Technologies) for measuring ICP and a Licox oxygen catheter microprobe (Integra Neuroscience) for measuring cerebral tissue oxygen pressure (PtiO_2_) were placed, after craniotomy, in the injured hemisphere. Continuous monitoring of mean arterial pressure, ICP and PtiO_2_ was performed for 4 h. At the end of the experiment, the rat was killed by decapitation under deep anesthesia.

### Brain Edema

Cerebral edema was measured by using a wet to dry weight technique, and results were expressed as a percentage of brain water content [19]. Sham rats and injured animals were killed at 2, 4, and 24 h post TBI by decapitation under deep anesthesia (*n* = 13 rats per group). The brains were then quickly removed, and both hemispheres were separated. Fresh injured hemispheres were immediately weighed to determine the wet weight and were placed at 105 °C for 48 h. Dehydrated structures were then reweighed to determine the dry weight. The brain water content was calculated as follows:

$${\text{Brain}}\,{\text{water}}\,{\text{content}} = \frac{{{\text{Wet}}\,{\text{weight}} - {\text{Dry}}\,{\text{weight}}}}{{{\text{Wet}}\,{\text{weight}}}} \times 100.$$

### Western Blotting

After decapitation (*n* = 5 rats per group), brains were rapidly removed, and both hemispheres were separated. Injured hemispheres were frozen in liquid nitrogen and smashed in a lysis buffer (20 mM HEPES, 0.4 M NaCl, 20% glycerol, 1% NP40, 5 mM MgCl_2_, 0.5 mM EDTA, and 0.1 mM EGTA). Tissues were lysed by 30-s sonication at 4 °C with an ultrasonic processor, and cell debris was removed by centrifugation (12,000 rpm for 45 min). Protein content was determined by using the Bradford protein assay [20]. Protein samples (100 μg) were separated with electrophoresis on 10% Tris–Cl polyacrylamide gel (100 V for 90 min at 4 °C). The resolved proteins were then transferred to a nitrocellulose membrane (electrophoresis, 80 V for 2 h at 4 °C). The blots were incubated in 5% nonfat dry milk and 0.1% Tween phosphate-buffered saline (PBS) solution for 90 min at room temperature and then incubated overnight at 4 °C with primary rabbit polyclonal antibodies (anti-AQP4 [Alomone ANT071AN0102, 1:500] and anti-KCC2 [Abcam AB49917, 1:500]). The blots were rinsed with PBS solution and incubated with horseradish peroxidase-conjugated secondary IgG (1:10,000) (reference) for 2 h at room temperature. Bound antibody was visualized by using the enhanced chemiluminescence assay and normalized with β-actin. Thus, we were able to study the time evolution (4 and 24 h) of AQP4 and KCC2 in the SL group.

### Immunofluorescence Staining

Anesthetized rats (*n* = 5 rats per group) were transcardially perfused with 4% paraformaldehyde in 0.01 M PBS. Brains were removed and then embedded in paraffin after fixation in 4% buffered paraformaldehyde and separation of both hemispheres. Serial 10-μm-thick coronal sections were prepared with microtome. The sections were deparaffinized and dehydrated with xylene and absolute ethyl alcohol for 5 min, washed three times with PBS, and incubated with 50 mM Tris–Cl (pH 8), 5 mM EDTA (pH 8), and 5 μg/μl proteinase K in 1 M PBS solution for 90 min at room temperature. Primary antibody samples (anti-AQP4 [Alomone ANT071AN0102, 1:200] and anti-KCC2 [Abcam AB49917, 1:150]) were incubated overnight at 4 °C in 2.5% goat serum. After being rinsed in PBS, slices were incubated with goat anti-rabbit Alexa 647 (1:400) for 1 h at room temperature. Fluorescence images were captured by a laser scanning confocal microscope (FV10i; Olympus). Western blot results were expressed as relative density compared to normalized protein expression in the Sham group (relative optical density = 100% in Sham group).

### Statistical Analysis

The Kolmogorov–Smirnov test confirmed the normal distribution of the experimental population. Data were expressed as mean ± standard deviation (SD). The comparison of two continuous variable groups was made with the Mann–Whitney *U* test. The comparison of three groups or more was made with the Kruskal–Wallis test, followed by a Dunn multiple comparison test (used when distribution is not normal). For abnormal distribution, we used an analysis of variance, followed by a Tukey multiple comparison test. A *P* value less than 0.05 was considered statistically significant.

## Results

### *Physiological Data: ICP and PtiO*_*2*_

**Fig. 1:**
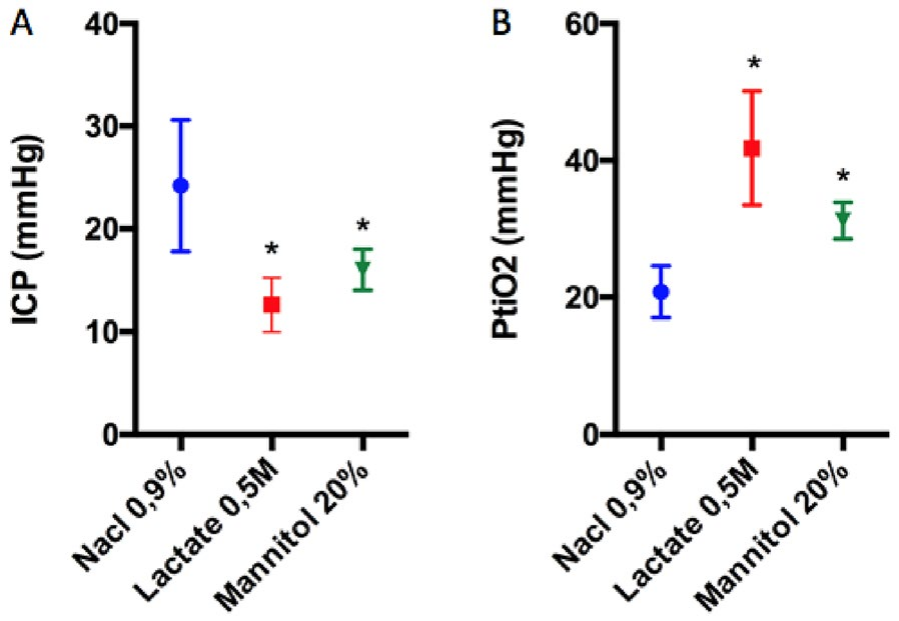
Effect of SL and mannitol. Effect of SL and mannitol on ICP (**a**) and PtiO_2_ (**b**) at 4 h post TBI. Values are expressed as mean ± SD. Asterisks indicate significant difference between vehicle and treated (mannitol or SL) TBI groups and other groups (*P* < 0.05) (*n* = 5 per group). ICP intracranial pressure, PtiO_2_ cerebral tissue oxygen pressure, SD standard deviation, SL sodium lactate, TBI traumatic brain injury

Both treatments with SL and mannitol decreased ICP from 24 to 12 mmHg (*P* = 0.02) and from 24 to 15 mmHg (*P* = 0.04), respectively. The magnitude of ICP reduction was not significantly different between the two treatments (*P* = 0.07) (Fig. 1a). Meanwhile, PtiO_2_ significantly increased by 90% in the SL group (from 21 to 40 mmHg; *P* = 0.008) and by 48% in the mannitol group (from 21 to 31 mmHg; *P* = 0.008) (Fig. 1b). No significant difference in PtiO_2_ was found between the SL group and the mannitol group (*P* = 0.06).

### Brain Water Content

**Fig. 2:**
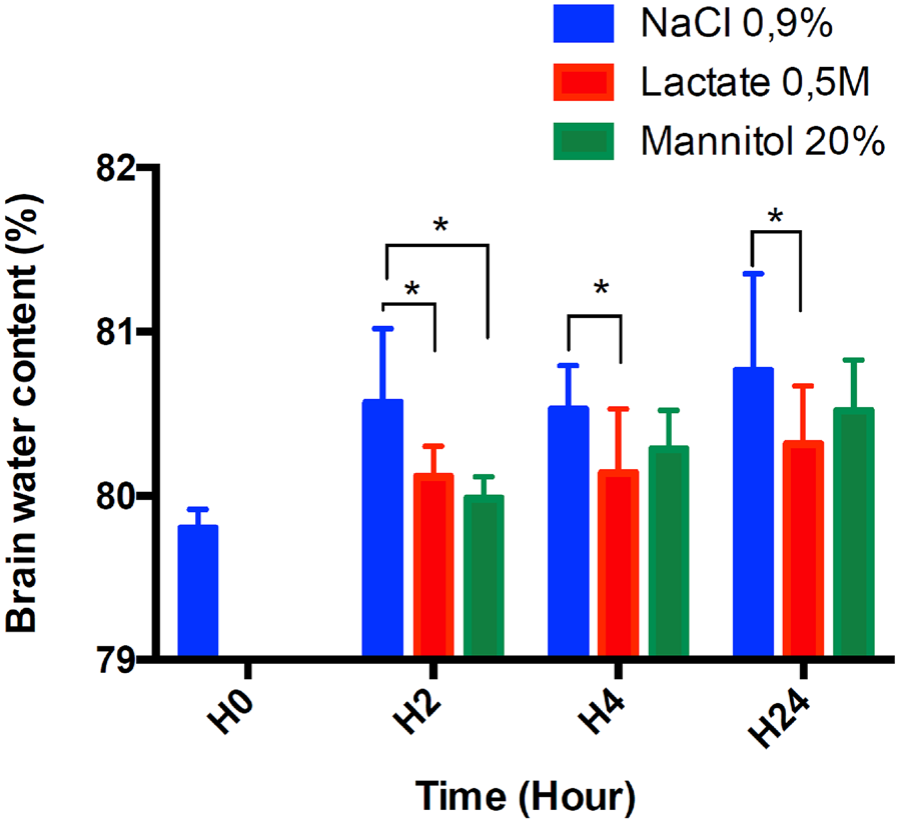
Effect of SL and mannitol on brain water content at different times after TBI. Values are expressed as mean ± SD. Asterisks indicate significant difference between vehicle and treated (SL or mannitol) TBI groups (*P* < 0.05) (*n* = 13 per group). SD standard deviation, SL sodium lactate, TBI traumatic brain injury

The brain water content in Sham-operated rats was calculated at 79.8% at baseline before injury. In the vehicle group, brain water content significantly increased during the first 24 h to 80.6% ± 0.5% at 2 h, 80.6% ± 0.3% at 4 h, and 80.8% ± 0.6% at 24 h (*P* < 0.05). In the mannitol group, brain water content significantly decreased only at 2 h post TBI (80.0% ± 0.1%; *P* < 0.05) (Fig. 2). The brain water content in the SL group showed a significant reduction at 2, 4, and 24 h compared with that in the vehicle group (*P* < 0.05; 80.1% ± 0.5%, 80.1% ± 0.4%, and 80.3% ± 0.4%, respectively).

### AQP4 Expression

**Fig. 3:**
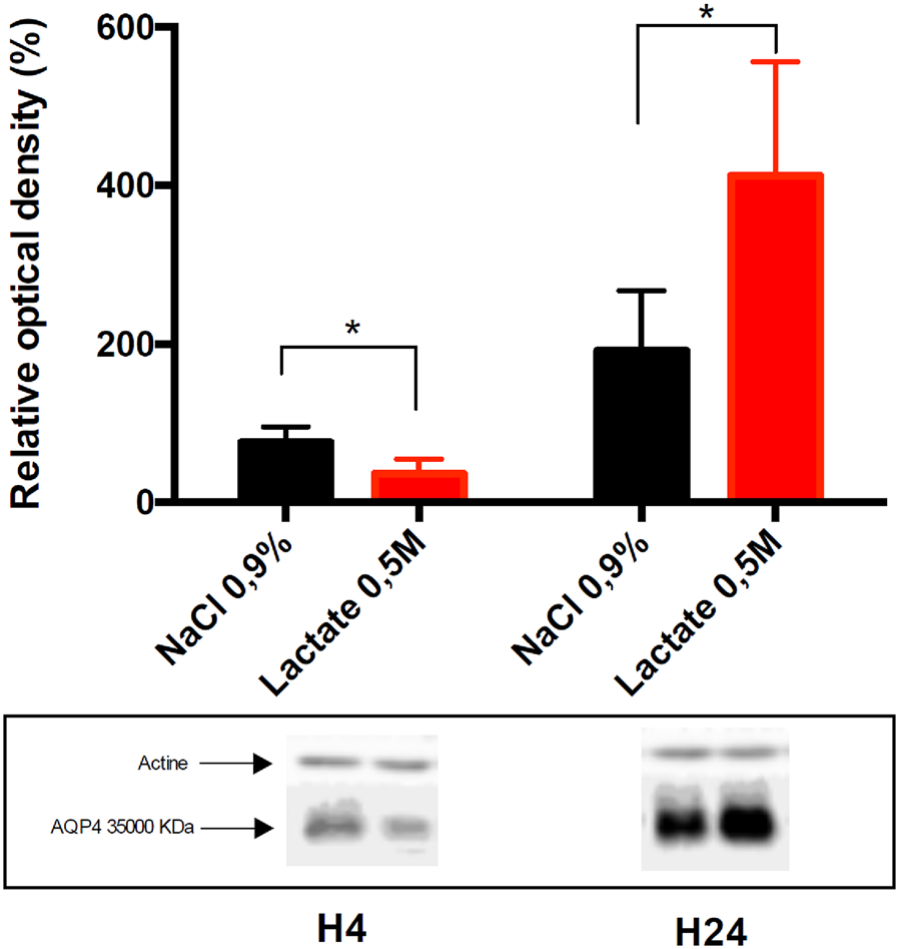
Western blot analysis of AQP4 protein expression in the ipsilateral injured cortex at 4 and 24 h post TBI in vehicle and SL groups. Relative density is presented as an increase percentage and compared with that of the Sham group. Values are expressed as mean ± SD. Asterisks indicate *P* < 0.05 (*n* = 5 per group). AQP4 aquaporin 4, SD standard deviation, SL sodium lactate, TBI traumatic brain injury

Western blot analyses showed that AQP4 protein expression was significantly reduced in the ipsilateral injured cortex at 4 h post TBI in the vehicle group compared with the Sham group (75.6% ± 36.14%; *P* < 0.05) (Fig. 3). SL induced more of a decrease in AQP4 expression (36.14% ± 8.4%; *P* < 0.05) in comparison with animals in the vehicle group (Fig. 3). However, AQP4 expression significantly increased at 24 h post TBI in the SL group compared with the vehicle group (412% ± 64.4% vs. 191.6% ± 33.7%, respectively; *P* < 0.05).

### KCC2 Expression

**Fig. 4:**
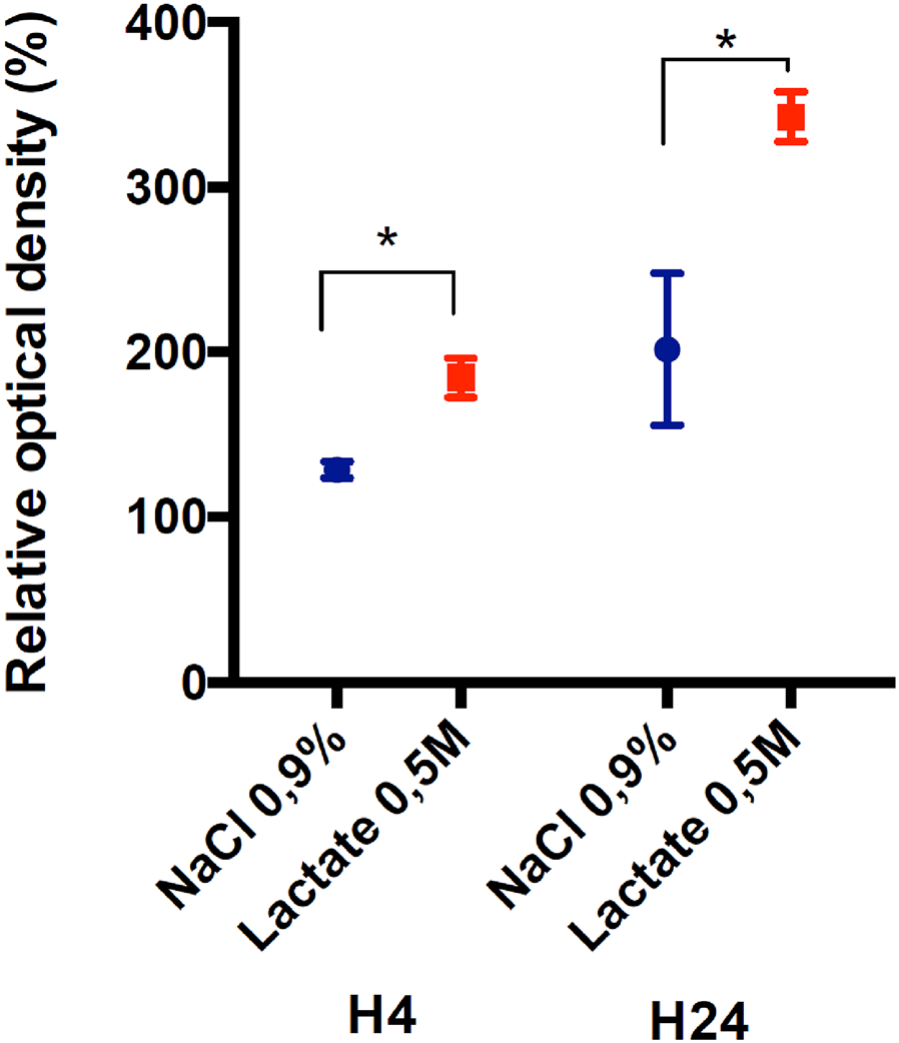
Western blot analysis of KCC2 expression after TBI plus vehicle treatment and TBI plus SL treatment. Relative density is presented as an increase percentage and compared with that of the Sham group. Values are expressed as mean ± SEM. Asterisks indicate *P* < 0.05 (*n* = 5 per group). SEM standard error of the mean, SL sodium lactate, TBI traumatic brain injury

**Fig. 5:**
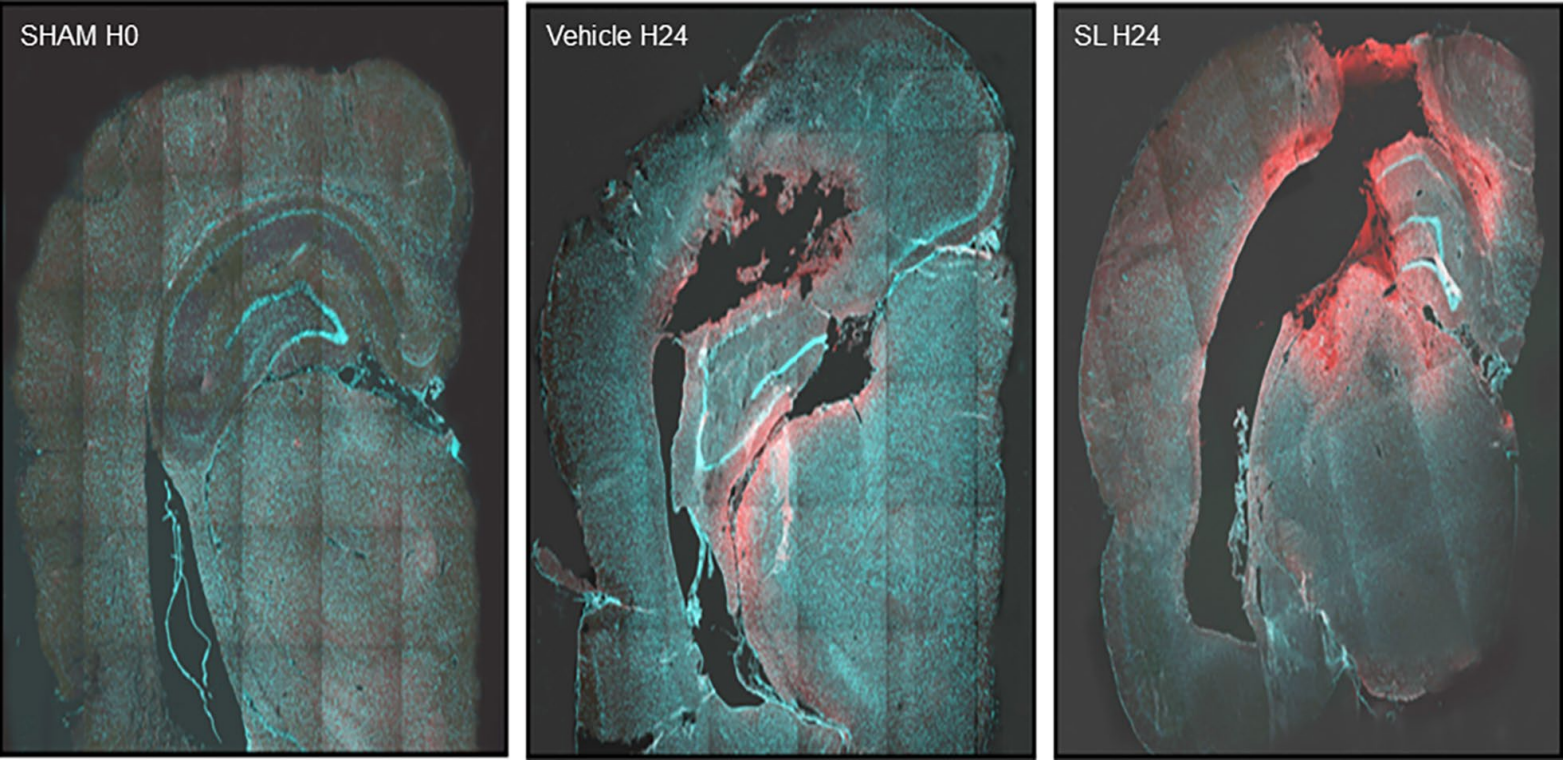
KCC2 immunolabeling in the Sham, vehicle, and SL groups. KCC2 immunolabeling (red) in the Sham group (left), in the vehicle group (middle) at 24 h post TBI, and in the SL group (right) at 24 h post TBI (10-µm sections) (*n* = 5 per group). SL sodium lactate, TBI traumatic brain injury (Color figure online)

KCC2 expression was analyzed at 4 and 24 h post TBI in the ipsilateral injured cortex. KCC2 expression in the vehicle group increased at 4 and 24 h after TBI induction (128.6% ± 2.2% and 201.6% ± 20.6%, respectively), whereas it did not change in the Sham group. SL treatment significantly increased the level of KCC2 protein expression at 4 and 24 h (184.4 ± 5.3 [*P* < 0.05] and 342 ± 6.8 [*P* < 0.05], respectively) in the comparison with the vehicle group (Fig. 4). Immunolabeling performed at 24 h after TBI showed that KCC2 was essentially located at the periphery of the contusional cortex of animals in the vehicle group and was strongly increased in the SL group (Fig. 5).

## Discussion

To our knowledge, this study is the first to demonstrate, in a TBI rat model, that exogenous SL administration is effective in reducing brain edema during elevated ICP episodes and is associated with improved brain oxygenation. Moreover, our data support a potential involvement of the chloride channel KCC and the AQP4 protein in this same context. This study seems to coincide with clinical data observed in previous studies. This observation is of importance because we could expect that in patients refractory to mannitol treatment, we could propose this alternative approach, using SL infusion, in an effort to reduce brain edema and improve cerebral oxygenation, based on a possible other way of action.

### The Effect of SL on ICP and Brain Water Content

In our results, a single bolus of half-molar SL induced a prolonged and persistent decrease of brain water content and ICP. Using equimolar doses, half-molar SL and 20% mannitol have a similar effect in reducing ICP, but the duration of the effect with mannitol is shorter. These results are consistent with previous clinical trials suggesting that half-molar lactate therapy was associated with a prolonged decrease in ICP, as compared with mannitol [4, 6]. Similar results were obtained with a continuous infusion of SL in a porcine model of TBI [21]. It is important to highlight the fact that the SL effect on brain water content persisted more than 24 h after trauma, whereas the effects of mannitol lasted for only a few hours. These results strengthen the hypothesis that the efficiency of hypertonic lactate therapy on cerebral edema cannot only be the result of a “classical osmotic effect consisting in an increased plasma hypertonicity,” as observed with mannitol [6]. This concept is strongly supported by previous clinical data in which it was reported that measured plasma osmolarity did not change after SL infusion, whereas it significantly increased after mannitol infusion. Because extracellular lactate easily penetrates into the cell because of its monocarboxylate transporters [22, 23], we can suggest that intracellular lactate is metabolized within the cell and that the remaining extracellular sodium is rapidly excreted in the urine, leading to a transient and nonrelevant change in plasma osmolarity. This is likewise supported by clinical data that indicates that an SL infusion in various conditions induces an increase in natriuresis [7, 24]. Furthermore, we may also conclude that because lactate penetrates easily into the cell, extrusion of anions would be necessary to maintain intracellular electroneutrality and equilibrium. The increased KCC2 expression could suggest that chloride is extruded probably to maintain this electroneutrality. Thus, the long-lasting effects of lactate on brain water content might be the result of the long-term regulation of KCC2.

### The Effects of SL on Brain Oxygenation

In the present work, we reported that both SL and mannitol increased PtiO_2_, a surrogate biological marker of improved brain oxygenation. However, this beneficial effect was more pronounced with SL than with mannitol therapy. Brain oxygenation has already been assessed in rodents with a diffuse TBI (that were then infused with mannitol) by associating a multiparametric magnetic resonance-based approach and direct measurements of cerebral oxygenation. The authors showed an improvement in brain tissue that might be the consequence of a reduction in astrocyte edema surrounding brain capillaries, allowing for better oxygen diffusion between microvessels and brain tissue [25]. On a structural analysis of post-TBI brain slices, it was observed that pericontusional ischemia is related to the compression of microvessels by swollen glial-foot processes [26]. By reducing edema in astrocytes, the main components of glial-foot processes, theoretically, brain oxygenation would be restored. Several other studies have also demonstrated a beneficial effect of SL on brain oxygenation. However, none of them allowed a precise determination as to the mechanism(s) involved. Both decreased astrocyte edema and lactate release are implicated in cerebral vessel diameter control [27, 28]. Indeed, lactate release from astrocytes into the extracellular space reduces the intracellular prostaglandin E2 transfer, leading to an extracellular accumulation of prostaglandin E2 and, in turn, cerebral artery vasodilation [27–29]. Finally, it can also be suggested that SL improves brain oxygenation as a consequence of improved brain metabolism by using lactate as an energy source. Such a mechanism is supported by numerous studies showing that lactate is a preferential and efficient substrate in the injured brain [4, 30].

### The Effects of SL on Cerebral AQP and Chloride Channel Expression

Among membrane proteins acting in the regulation of cerebral edema, AQPs are considered as the main channels for free water transport [15]. AQPs are hydrophobic, intrinsic membrane proteins composed of two transmembrane domains and two intramembrane domains [31]. They allow bidirectional passive diffusion of water. Of the 14 subtypes identified, only AQP forms 1, 4, and 9 are expressed in the central nervous system. Under physiological conditions, AQP4 is mainly expressed in the astrocytic foot that forms the outer layer of the blood–brain barrier. After trauma, AQP4 spreads along the astrocytic membrane [32]. The role of AQP4 is to neutralize the osmotic gradients related to ion channel activation by facilitating free water diffusion across the cell membrane. Early inhibition of AQP4 expression has been shown to reduce brain edema and improve posttraumatic recovery [33]. However, the protective or deleterious role of AQP4 in cerebral edema regulation remains unclear. AQP4 allows the elimination of extracellular water during vasogenic edema and regulates some inflammatory processes during the late phase of brain edema. On the other hand, AQP4 also increases cellular edema in the early phase [16, 34, 35]. For these reasons, we focused our study on describing in more detail the role of AQP4 in TBI. In the present study, SL infusion seems to regulate AQP4 expression in two opposite ways depending on the timing from brain injury: AQP4 expression was significantly reduced in the comparison with animals in the vehicle group in the early phase but, on the contrary, overexpressed in the later phase. Such a biphasic evolution might promote a reduction in brain edema in the early phase and water clearance in the later stage. The beneficial effects of AQP4 involvement in the improvement of TBI-induced deficits is consistent with recent studies on neuroprotective therapy using traditional Chinese medicine in an animal model of TBI [36].

It would seem to follow that a regulatory volume decrease mechanism is thus activated to prevent the development of cell swelling following TBI. It has already been suggested that a cellular loss of chloride via an activation of the K–Cl cotransporters plays a major role in this phenomenon [9, 37]. Cell swelling inhibits WNK-SPAK/OSR1 kinase and stimulates protein phosphatases that dephosphorylate NKCC and the KCCs, causing their inhibition and activation, respectively. The resulting net ionic efflux of potassium and chloride, coupled with obligatory water movement, restores cell volume [38]. It has already been reported that cortical neurons lacking KCC2 expression are unable to regulate intracellular chloride concentrations [12]. Our immunohistological data support the involvement of CCCs in the antiedematous effect of hypertonic SL. KCC2 overexpression after TBI might be the cellular response to restore normal cell volume after brain cell swelling. However, considering the 24-h delay for these changes to take place, KCC2 overexpression might not be the first trigger for cell volume regulation induced by lactate therapy, but rather one piece of a more complex regulation, possibly a genomic mechanism. Indeed, KCC2 overexpression requires time to be effective and might essentially be involved in a long-term regulatory action. Short-term cell volume regulation is usually accomplished by the activation of plasma membrane channels, such as volume-regulated anion channels (VRACs) [39, 40]. In this way, an interaction between VRACs and KCCs is suggested in some studies, showing that mutations in KCCs can induce brain disorders associated with intracellular swelling [38].

Our results, however, must be tempered. Despite our physiological data being in line with clinical data previously reported [6, 7], our experimental data issuing from a model of rat TBI must be cautiously extrapolated to human head injury. However, we could expect that the use of a mixed model of experimental TBI, as LFPI, would have limited this bias. We decided also not to assess the impact of SL administration on functional parameters, such as the neurological outcome, and especially cognitive performances because in previous data, a significant effect related to lactate perfusion and neurological recovery has already been reported. Secondly, the effect of a continuous perfusion instead of a bolus dose was not investigated. Further investigations are required to determine the most effective modality for SL administration in this condition. Thirdly, a beneficial effect related to the use of SL as a source of energy cannot be eliminated, and we could expect that metabolic mechanisms and antiedematous mechanisms are probably associated. A better understanding of intracellular lactate metabolism is necessary to explore this concept. Fourthly, the comparison with HS may need to be questioned. However, as previously reported, SL did not significantly increase plasma osmolarity, whereas the contrary was the case with HS [1, 7, 41]. Moreover, lactate by itself has been found to have various beneficial effects in brain injury, including vasodilation, the disposition of an energetic substrate, and a decrease in intracellular tonicity leading to a extrusion of both anions (especially chloride) and water [37]. A comparison of SL and HS remains, however, necessary to eliminate the role of sodium as a possible component of the SL effect. Finally, we have attempted to elicit some of the potential interactions associated with chloride channels and brain edema. However, such a phenomenon requires confirmation by inhibiting the expression of chloride channels and then assessment of the evolution of brain edema. A protocol focused on the inhibition of VRACs in a TBI model, is already in progress.

## Conclusions

In conclusion, this is the first preclinical study designed to confirm the antiedematous effect of exogenous SL administration on brain tissue post TBI and to investigate the possible mechanisms involved in this effect. Half-molar SL infusion strongly reduces cerebral edema and improves brain oxygenation. We provide further evidence that the antiedematous action of SL could be correlated with chloride channel regulation. Compared with mannitol, SL seems to induce more complex mechanisms than that centering on a purely osmotic effect. We report for the first time the potential role of chloride channels in reducing brain edema. Further large experimental and clinical studies are required to validate the observations and confirm SL as an alternative therapeutic option for the management of patients with severe TBI.
